# Supplementary material for: An herbal drug combination identified by knowledge graph alleviates the clinical symptoms of plasma cell mastitis patients: A nonrandomized controlled trial
Source: eLife. 2023 Mar 14;12:e84414. doi: 10.7554/eLife.84414 (PMC10063228; doi:10.7554/eLife.84414)
Supplement: Supplementary file 1. — (A) Baseline characteristics of patients in the clinical trial. (B) Clinical symptom rating scale for plasma cell mastitis (PCM). [file elife-84414-supp1.docx]

**Supplementary File 1**

**An herbal drug combination identified by knowledge graph alleviates the clinical symptoms of plasma cell mastitis patients: a nonrandomized controlled trial**

Caigang Liu^1, *^, Hong Yu^1, #^, Guanglei Chen^1, #^, Qichao Yang^2^, Zichu Wang^2^, Nan Niu^1^, Ling Han^3^, Dongyu Zhao^4^, Manji Wang^5, *^, Yuanyuan Liu^6^, Yongliang Yang^2, *^,

^1^*Cancer Stem Cell and Translation Medicine Lab, Innovative Cancer Drug Research and Development Engineering Center of Liaoning Province, Department of Oncology, Shengjing Hospital of China Medical University, Shenyang, 110004, China*;

^2^School of Bioengineering, Dalian University of Technology, Dalian, 116023, China;

^3^*National Engineering Research Center of Pharmaceutics of Traditional Chinese Medicine*, *China Resources Sanjiu Medical & Pharmaceutical Co., Ltd*, *Shenzhen*, *China*;

^4^*International Cancer Institute, Peking University Health Science Center, Peking University*, *Beijing*, *100191*, *China*;

^5^Shanghai BeautMed Corporation, Shanghai, 200030, China;

^6^Department of Biology, University of Copenhagen, Denmark.

**List of Contents**

1. **Baseline characteristics of plasma cell mastitis (PCM) patients**
2. **Clinical symptom rating scale for plasma cell mastitis (PCM)**

**Supplemental File 1A. Baseline characteristics of patients in the clinical trial.**

| **Item** | **EG（n=80）** | **CG（n=80）** | **t/X^2^** | **P** |
| --- | --- | --- | --- | --- |
| Age（years） | 32.87±5.07 | 32.25±5.45 | 0.451 | 0.653 |
| Height（cm） | 162.87±4.63 | 161.94±5.09 | 1.212 | 0.227 |
| Body weight（kg） | 69.20±14.10 | 66.46±13.28 | 1.262 | 0.209 |
| Body mass index（kg/㎡） | 26.17±5.70 | 25.36±5.08 | 0.946 | 0.346 |
| Childbearing age（years） | 28.50±3.53 | 28.08±3.27 | 0.758 | 0.450 |
| Clinical classification |  |  | 1.829 | 0.401 |
| Fistula type | 10 | 15 |  |  |
| Mass type | 45 | 46 |  |  |
| Mass type | 25 | 19 |  |  |
| Breastfeeding |  |  | 1.493 | 0.222 |
| Yes | 73 | 68 |  |  |
| No | 7 | 12 |  |  |
| History of mastitis |  |  | 0.233 | 0.629 |
| Yes | 46 | 49 |  |  |
| No | 34 | 31 |  |  |
| Nipple state |  |  | 0.119 | 0.730 |
| Normal | 57 | 55 |  |  |
| Invagination | 23 | 25 |  |  |
| History of breast trauma |  |  | 0.406 | 0.524 |
| Yes | 33 | 37 |  |  |
| No | 47 | 43 |  |  |
| The fetus gender |  |  | 0.36 | 0.627 |
| Male | 30 | 33 |  |  |
| Female | 50 | 47 |  |  |

**Supplemental File 1B. Clinical symptom rating scale for plasma cell mastitis (PCM).**

| **Item** | **0** | **2** | **4** | **6** |
| --- | --- | --- | --- | --- |
| Mass size | No | ≤2cm×2cm | ＞2cm×2cm and ≤5cm×5cm | >5cm×5cm |
| Swelling | No | ≤2cm×2cm | ＞2cm×2cm and ≤5cm×5cm | >5cm×5cm |
| Discharge | No | Mild (clear as green water sample) | Medium (thick and milky) | Severe (viscous, lipid-like) |
| Retraction | No | Mild depression | Completely depressed, can return to normal after stimulation | Completely depressed, cannot return to normal after stimulation |
| Abscess | No | Not burst | Brust | Brus repeatedly |
| Fistula | No | Single fistula | Double fistula | Many fistula |
