# Supplementary material for: An herbal drug combination identified by knowledge graph alleviates the clinical symptoms of plasma cell mastitis patients: A nonrandomized controlled trial
Source: eLife. 2023 Mar 14;12:e84414. doi: 10.7554/eLife.84414 (PMC10063228; doi:10.7554/eLife.84414)
Supplement: Supplementary file 2. [file elife-84414-supp2.docx]

**A single arm and single center clinical study on the treatment of plasma cell mastitis with traditional Chinese medicine**

**Study Code** Shengjing-LCG012

**Version NO.** 1.1

**Version date** 18 Feb. 2022

**Study Site** Shengjing Hospital Affiliated to China Medical University

**Principal investigator** Caigang Liu

**Study Coordinating Site**

| **Information** | |
| --- | --- |
| **Site Name** | Shengjing Hospital Affiliated to China Medical University |
| **Site Address** | 39 Huaxiang Road, Tiexi District, Shenyang 110021 |
| **Principal investigator** | Caigang Liu |

**Signature page**

**Signature of the researcher**

I will conscientiously perform the duties as a researcher in accordance with the Chinese GCP regulations, and participate in or guide this clinical study. I hereby confirm that I have read this scheme (version number: 1.1; version date: February 18, 2022). I agree to perform relevant duties in accordance with the Chinese law, the Declaration of Helsinki, and the China GCP. This research proposal would not be implemented without the consent of the Ethics Committee, unless measures should be taken for the safety, rights and interests of the subjects.

**Research unit： Shengjing Hospital Affiliated to China Medical University**

**LiuCaigang
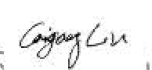
**February 18. 2022

**Principalinvestigator(PrintBody) Signature Date (year/month/day)**

# SYNOPSIS

| **Study Title** | A single arm and single center clinical study on the treatment of plasma cell mastitis with traditional Chinese medicine |
| --- | --- |
| **Study Drugs** | Traditional herbal medicine for immunotherapy |
| **Background** | Plasma cell mastitis (PCM), also known as Mammary Ductectasia (MDE), is a chronic non-bacterial breast inflammation with ductal dilatation and plasma cell infiltration as the basis. According to reports at home and abroad, the incidence of PCM is about 1.41% ~ 5.36% of benign breast diseases.  The etiology of plasma cell mastitis has not been definitively determined. It is generally believed to be related to breast structural abnormalities such as nipple invagination, breast trauma, bacterial infection, increased secretion of prolactin, use of birth control pills, smoking and other factors. However, recent studies have shown that plasma cell mastitis belongs to the category of autoimmune disease, because CD3, CD20 and CD45 positive cells can be detected in the lobules, ducts and around the interstitium of mammary glands of plasma cell mastitis patients, and a large number of lymphocytes are infiltrated, in addition, a small number of CD68-positive macrophages are infiltrated. Therefore, PCM is a new autoimmune benign breast disease which is different from common mastitis and breast diseases.  PCM is more common in young and middle-aged women, with a high incidence age of 30-40 years, and occasionally in men and children. Mammary gland mass can be long-term stable existence, but also secondary acute bacteria and (or) anaerobic bacteria infection, local redness and swelling heat pain is obvious, the formation of abscess. On this basis can be secondary abscess broken ulceration, long unhealed sinus tract formation. Although this disease is an inflammatory lesion, the effect of conventional drug treatment is not good, and it is still easy to relapse after multiple operations. The abscess repeatedly breaks and forms sinus tract, fistula or prolonged unhealed ulcer, which seriously affects the appearance of the breast and the quality of life of patients.  In order to solve the pain of patients, the Cancer Center of Shengjing Hospital Affiliated to China Medical University and experts from the Department of traditional Chinese Medicine formed a research team to explore the effects of plasma cell mastitis on autoimmunity and its mechanism. The effective and non-toxic formulations of traditional Chinese medicine and medicine and food homology - Sanyongjiedu prescription and Sanyongfuzheng prescription were formulated. Prescription is targeted at patients with different stages of disease, precise treatment, effectively shorten the course of disease, reduce the pain of patients, so that the appearance of the breast is not greatly affected. |
| **Subjects** | 50 patients with plasma cell mastitis were enrolled. |
| **Study**  **Objectives** | To explore the efficacy, immune function and quality of life of plasma cell mastitis treated with traditional herbal medicine |
| **StudyEndpoints** | Clinical cure rate; Clinical response rate; Operation rate; Recurrence rate. Incidence of adverse events |
| **Overall Study**  **Design** | Traditional Chinese medicine prescriptions include two kinds of prescriptions, Sanyongjiadu granules (for acute phase) and Sanyongfuzheng granules (for remission phase). Different treatments are given according to the clinical manifestations and stages of the subjects, and the prescriptions are adjusted according to the clinical manifestations. Acute onset, mainly breast mass, given the carbuncle Sanyongjiedu particles; For a long course of disease, the formation of mammary abscess after the delivery of Sanyongfuzheng granules; If the breast mass with abscess formation, according to the patient's onset time and specific clinical manifestations to give medication.  At the initial stage of treatment, the patient was revisited every 15 days. Return once a month after the condition has improved significantly; Through physical examination, biochemical examination and breast ultrasound, the therapeutic effect of Sanyongjiedu granules and Sanyongfuzheng granules on plasma cell mastitis was evaluated, and the general and clinical information of subjects with effective treatment and those without treatment were compared to find out the risk factors affecting the prognosis of plasma cell mastitis.  **Evaluation criterion:**  TCM Symptom score  Pain score  Quality of life score  Blood tests:C-reactive protein;Cytokines;Lymphocyte subsets;Tumor necrosis factor；  Interferon  **The overall design of this study is as follows:**  **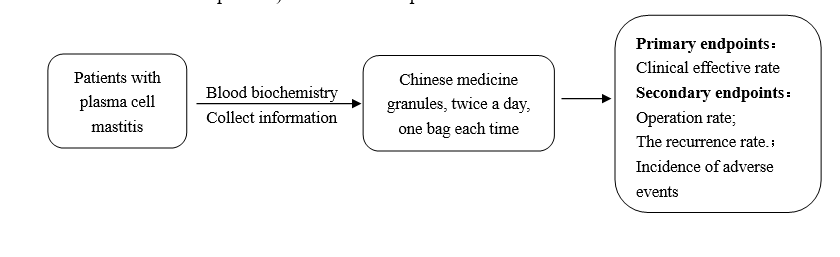** |
| **Inclusion Criteria** | Subjects must meet all the following inclusion criteria to be enrolled in this trial:  1. Female patients aged ≥ 20 but ≤ 60 years;  2. Meet the diagnostic criteria for plasma cell mastitis in Western medicine and traditional Chinese medicine；  3. The subject's informed consent, voluntary subjecting and signing of the informed consent form |
| **Exclusion Criteria** | Anyone who has one of the following conditions cannot participate in the clinical study:  1. Pregnant, lactating women or those who are unwilling to take contraception during the trial period and within 1 month after the end of the trial;;  2. Patients with serious underlying diseases, such as diabetes, cardiovascular and cerebrovascular diseases, liver, kidney and hematopoietic system diseases;;  3. Allergic constitution, such as a history of allergies to two or more drugs or foods; or those who are known to be allergic to the ingredients of the drugs used in this test;;  4. Recent use of antidepressants and other psychotropic drugs;  5. Hormone and immunosuppressive therapy were used one month before enrollment;  6. Poor compliance cannot cooperate to complete the test; |
| **Study**  **Procedures** | The patients were given sanyongjiedu granules, specific prescriptions: dandelion 15g, forsythiae 15g, honeysuckle 10g, Runlu 8g, purple flower diding 20g, salvia miltiorrhiza 10g, astragalus membranaceus 20g, licorice 8g; For abscess rupture or sinus formation of patients with sanyongFuzheng particles, specific groups of astragalus 20g, angelica 10g, ginseng 8g, honeysuckle 10g, dandelion 15g, forsythiae 15g, trichosanthin 12g, processed licorice 8g; The above-mentioned drugs were provided by Shengjing Hospital Affiliated to China Medical University. All of them were avoid fried particles, which were administered once in the morning and once in the evening for 2 months. |
| **Statistical analysis** | SPSS statistical software was used to process the data. Independent sample t-test or  analysis of variance was used for the measurement data (± s), and *x*test was used for  the count data (%). *P*<0.05 was statistically significant. |
| **Study Schedule** | Anticipated enrollment of the first subject: October, 2021  Anticipated enrollment of the last subject: October, 2022 |

1. **Research Background**

Plasma cell mastitis (PCM), also known as Mammary Ductectasia (MDE), is a chronic non-bacterial breast inflammation with ductal dilatation and plasma cell infiltration as the basis. According to reports at home and abroad, the incidence of PCM is about 1.41% ~ 5.36% of benign breast diseases.

The etiology of plasma cell mastitis has not been definitively determined. It is generally believed to be related to breast structural abnormalities such as nipple invagination, breast trauma, bacterial infection, increased secretion of prolactin, use of birth control pills, smoking and other factors. However, recent studies have shown that plasma cell mastitis belongs to the category of autoimmune disease, because CD3, CD20 and CD45 positive cells can be detected in the lobules, ducts and around the interstitium of mammary glands of plasma cell mastitis patients, and a large number of lymphocytes are infiltrated, in addition, a small number of CD68-positive macrophages are infiltrated. Therefore, PCM is a new autoimmune benign breast disease which is different from common mastitis and breast diseases.

PCM is more common in young and middle-aged women, with a high incidence age of 30-40 years, and occasionally in men and children. Mammary gland mass can be long-term stable existence, but also secondary acute bacteria and (or) anaerobic bacteria infection, local redness and swelling heat pain is obvious, the formation of abscess. On this basis can be secondary abscess broken ulceration, long unhealed sinus tract formation. Although this disease is an inflammatory lesion, the effect of conventional drug treatment is not good, and it is still easy to relapse after multiple operations. The abscess repeatedly breaks and forms sinus tract, fistula or prolonged unhealed ulcer, which seriously affects the appearance of the breast and the quality of life of patients.

In order to solve the pain of patients, the Cancer Center of Shengjing Hospital Affiliated to China Medical University and experts from the Department of traditional Chinese Medicine formed a research team to explore the effects of plasma cell mastitis on autoimmunity and its mechanism. The effective and non-toxic formulations of traditional Chinese medicine and medicine and food homology - Sanyongjiedu prescription and Sanyongfuzheng prescription were formulated. Prescription is targeted at patients with different stages of disease, precise treatment, effectively shorten the course of disease, reduce the pain of patients, so that the appearance of the breast is not greatly affected.

# 2.Study objectives and endpoints

| Research purposes | Study endpoint |
| --- | --- |
| The main purpose | Primary endpoint |
| To explore the efficacy, immune function and quality of life of patients with plasma cell mastitis treated with traditional herbal medicine | Clinical effective rate |
|  | Secondary endpoints |
|  | Operation rate;  The recurrence rate.；  Incidence of adverse events |

**3.Research design**

Traditional Chinese medicine prescriptions include two kinds of prescriptions, Sanyongjiadu granules (for acute phase) and Sanyongfuzheng granules (for remission phase). Different treatments are given according to the clinical manifestations and stages of the subjects, and the prescriptions are adjusted according to the clinical manifestations. Acute onset, mainly breast mass, given the carbuncle jiedu particles; For a long course of disease, the formation of mammary abscess after the delivery of sanjiu Fuzheng granules; If the breast mass with abscess formation, according to the patient's onset time and specific clinical manifestations to give medication.

At the initial stage of treatment, the patient was revisited every 15 days. Return once a month after the condition has improved significantly; Through physical examination, biochemical examination and breast ultrasound, the therapeutic effect of Banyongjiedu granules and Sanyongfuzheng granules on plasmacytic mastitis was evaluated, and the general and clinical information of subjects with effective treatment and those without treatment were compared to find out the risk factors affecting the prognosis of plasmacytic mastitis.

**The overall design of this study is as follows：**

**
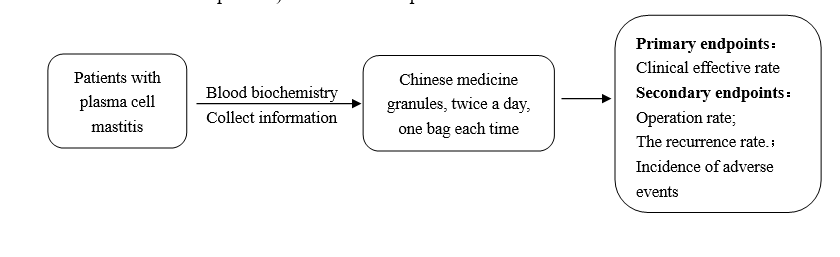
**

# Subject selection and withdrawal

## 4.1 Inclusion criteria

Subjects must meet all of the following inclusion criteria to be enrolled in this trial:

1. Female patients aged ≥ 20 but ≤ 60 years;
2. Meet the diagnostic criteria for plasma cell mastitis in Western medicine and traditional Chinese medicine；

Diagnostic criteria of traditional Chinese medicine

(1) Liver heat stasis syndrome: breast agglomeration, redness, swelling and pain, accompanied by fever, headache, dry stool, yellow urine, red tongue, yellow and greasy tongue coating, pulse string number or slip number.

(2) the syndrome of stagnation of deficiency: abscess self-ulceration or incision after a long time, pus dripping, the formation of milk leakage, more and more hair, local stiff mass, tongue reddish or red, thin yellow coating, pulse string

Diagnostic criteria of Western medicine

(1) Most of the cases occurred in non-lactation or non-pregnancy women, unilateral breast incidence was more common, but also bilateral incidence;

(2) Most of the congenital nipple with all or part of the depression;

(3) areola mass, or to a quadrant extension, diameter of one to a few centimeters, irregular shape, hard and tough, nodular surface, boundary is not clear, often with skin adhesion, but no chest wall fixed, can occur redness and pain, sometimes the mass is not redness and swelling, some are orange peel like change, affected side axillary lymph node enlargement tenderness;

(4) Fine needle aspiration cytology of the mass showed a large number of plasma cells.

1. The subject's informed consent, voluntary subjecting and signing of the informed consent form

**4.2 Exclusion Criteria:**

Anyone who has one of the following conditions cannot participate in the clinical study:

1. Pregnant, lactating women or those who are unwilling to take contraception during the trial period and within 1 month after the end of the trial;;

2. Patients with serious underlying diseases, such as diabetes, cardiovascular and cerebrovascular diseases, liver, kidney and hematopoietic system diseases;;

3. Allergic constitution, such as a history of allergies to two or more drugs or foods; or those who are known to be allergic to the ingredients of the drugs used in this test;;

4. Recent use of antidepressants and other psychotropic drugs;

5. Hormone and immunosuppressive therapy were used one month before enrollment;

6. Poor compliance cannot cooperate to complete the test;

## 4.3 Withdrawal Criteria

**4.3.1 Criteria for subject withdrawal**

1. The subject voluntarily withdraws the informed consent at any time;

2. After randomization, it was found that the subjects seriously violated the inclusion and exclusion criteria;

3. Medical imaging progress or clinical progress;

4. There are any clinical adverse events, abnormal laboratory tests or other medical conditions, which may cause the subjects

to continue to use the drug and may no longer benefit;

5. During the course of the research, the subject has a pregnancy event;

6. Serious violation of the trial protocol, and the investigator's assessment believes that the treatment should be terminated;

7. Other reasons considered by the investigator to be unable to continue the study drug treatment.

**4.3.2 Handling of withdrawal subjects**

Investigators can suggest or provide new or alternative treatments to patients based on their actual conditions.

## 4.4 Termination criteria

The study termination criteria include but are not limited to the following:

1. Unexpected, significant or unacceptable risks to the subject are found;

2. Major mistakes were found in the plan during the execution of the test;

3. The study drug/trial treatment is ineffective, or it is meaningless to continue the trial;

4. Completion of the trial is extremely difficult due to reasons such as severe delays in subject selection or frequent protocol

deviations.

If the principal investigator terminates or suspends the clinical study in advance, the subjects must be informed immediately,

and the ethics committee shall be reported in writing with the specific reasons.

# Study drug

## 5.1 Study drug name and source

1. Treatment drugs for acute phase (Sanyongjiedu granules)

[Prescription composition] Dandelion 15g, Forsythia 15g, Honeysuckle 10g, Ruanlu 8g,Purple flower diddin 20g, Salvia miltiorrhiza 10g, Astragalus 20g, Licorice 8g

2. Medication for chronic phase (Sanyongfuzheng granules)

[Prescription composition] Astragalus 20g, Angelica 10g, Ginseng 8g, Honeysuckle 10g,Dandelion 15g, Forsythia 15g, Trichosanthin 12g, Processed licorice 8g

## 5.2 Method of administration

# Traditional herbal medicine for cancer immunotherapy: twice a day (continuous);

# Two kinds of prescriptions are decoction free.

# Usage: boiled water, 1 bag at a time, 2 times a day, avoid menstrual period, avoid spicy stimulation, avoid eat cold food, avoid emotional or sulking.

**5.3 Treatment of adverse events**

The component oftraditional herbal medicine is drug food homologous. It refers to the "absolutely safe" traditional Chinese medicine defined by the National Health Commission Therefore, there will be no adverse events in theory. In case of adverse events during the study, the researcher will actively deal with the symptoms according to clinical routine procedures, and make detailed records. At the same time, individual differences should be considered. Patients with allergic constitution are prone to cause drug allergic reactions, also known as drug allergic reactions. In addition, special attention should be paid.

# 6 Study procedures

Patients must read and sign a current Ethics Committee (EC)-approved informed consent form before starting the study. All research steps need to be carried out within the time window indicated in the research schedule.

## 6.1 Screening period

Collect general information of the subjects, including: Drinking age, height, weight, smoking history, menstrual, examination and nursing of lactation mastitis (history), history of basic diseases (hypertension, diabetes, heart disease and other medical or other malignant tumor history), a history of mental illness (e.g., postpartum depression, etc.), mammary gland injury history, nipple anatomical morphology (normal or invagination), marital status, Pictures before and after medication were kept.

## 6.2 Treatment period

Blood routine, cytokines (IL and TNF), lymphocyte subsets, IgA and IgG before and after treatment were were collected; Different treatments were given according to the clinical manifestations and clinical stages of the subjects, and the prescriptions were adjusted according to the clinical manifestations of the subjects. Acute onset, mainly breast mass, given the sanyongjiedu particles; For a long course of disease, the formation of mammary abscess after the delivery of sanyongfuzheng granules; If the breast mass with abscess formation, according to the patient's onset time and specific clinical manifestations to give medication.

At the initial stage of treatment, the patient was revisited every 15 days; Return every 30 days after significant improvement; The patients were followed up for more than 6 months after cure. By comparing the general and clinical information of patients with effective and ineffective treatment, the risk factors affecting the prognosis of plasma cell mastitis were found.

## 6.3 Combined treatment provisions

## 1. Antibiotic therapy: Decide whether it is necessary according to the condition.

## 2.Hormone therapy: Decide whether it is necessary according to the condition

## 3.Anti-tuberculous drug treatment: decide whether it is necessary according to the condition

## 4.Other drug treatment: Decide whether it is necessary according to the condition

## 5.Surgical treatment: Decide whether it is necessary according to the condition6.4 End of study treatment/withdrawal from study

## 6.4 Follow-up

## The follow-up was divided into treatment period follow-up and follow-up. During the treatment, the patients were followed up every 15 days. Return every 30 days after significant improvement; Patients were followed up for more than 6 months after the disease was cured. The follow-up included medication records, adverse events, clinical symptom score, biochemical examination, quality of life score and pain score.

# 7 Effectiveness evaluation

The therapeutic effect was determined according to the "Criteria for Diagnosis and Therapeutic Effect of Diseases in Traditional Chinese Medicine" issued by the State Administration of Traditional Chinese Medicine. After treatment, all clinical symptoms disappeared, the skin returned to normal, and the mass disappeared, and the patient was judged to be cured. After treatment, the clinical symptoms of the patients were significantly improved, and the mass was reduced. After treatment, the patient's various clinical symptoms were not significantly improved, or even aggravated, and was judged to be ineffective. Total effective rate = (number of cured patients + number of effective patients)/number of total patients ×100%.

## 7.1 Clinical symptom scores

By the state administration of traditional Chinese medicine clinical symptom assessment with reference to the disease diagnosis curative effect of traditional Chinese medicine standard and the Chinese medicine new medicine clinical research guiding principles (try out) , the formulation of the PCM clinical symptoms scale(Table 1),comparing before and after treatment in patients with clinical signs.

Table1 Clinical symptom rating scale of plasma cell mastitis

| Item | 0 | 2 | 4 | 6 |
| --- | --- | --- | --- | --- |
| Mass size | No | ≤2cm×2cm | ＞2cm×2cm and ≤5cm×5cm | >5cm×5cm |
| Swelling | No | ≤2cm×2cm | ＞2cm×2cm and ≤5cm×5cm | >5cm×5cm |
| Discharge | No | Mild (clear as green water sample) | Medium (thick and milky) | Severe (viscous, lipid-like) |
| Retraction | No | Mild depression | Completely depressed, can return to normal after stimulation | Completely depressed, can not return to normal after stimulation |
| Abscess | No | Not burst | Brust | Brus repeatedly |
| Fistula | No | Single fistula | Double fistula | Many fistula |

## 7.2 Painscores

Pain assessment method used the oral description score , which was divided into 1 for no pain, 2 for very mild, 3 for mild, 4 for serious, 5 for severe, and 6 for very severe. The pain scores before and after treatment were observed and compared.

## 7.3 Quality of life scores

The quality of life rating scale was developed according to the quality of life evaluation scale SF-36 . The higher the score,

the better the quality of life. The quality of life scores of patients before and after treatment were observed and compared.

| **Quality of life scores**  Note that all the questions are only about your last month. Please read each question and choose the answer that best suits your situation according to your feelings. |
| --- |
| 1. How do you evaluate your quality of life? □ Very bad □ Bad □ Not good and not bad □ Good □ Very good  2. Are you satisfied with your health? □ Not satisfied □ Not satisfied □ Neither satisfied nor dissatisfied □ Satisfied □ Very satisfied  3. Do you feel that pain prevents you from doing what you need to do?  □ Not at all □ Rarely □ Somewhat (normal) □ Relatively □ Very much  4. Do you need medical help to go about your daily life? □ Not required □ Rarely required □ Required (normal) □ Relatively required □ Extremely required  5. Do you find life enjoyable? □ No fun □ Little fun □ Fun (normal) □ More fun □ Lots of fun  6. Do you feel your life has meaning? □ No meaning □ Little meaning □ Meaningful (normal) □ Relatively meaningful □ Very meaningful  7. Can you pay attention? □ Not at all □ Few □ Energy (average) □ Relatively energy □ Extreme energy  8. Do you feel safe in your daily life? □ Not secure □ Rarely secure □ Secure (normal) □ Relatively secure □ Extremely secure  9. Is your living environment healthy? □ Not good □ Rarely good □ Good (average) good □ Excellent  10. Do you have enough energy to cope with daily life?  □ No energy □ Little energy □ Energy (average) □ Most energy □ Total energy  11. Do you think your appearance is passable? □ It is impossible to pass □ Rarely pass □ Pass (fair) □ Mostly pass □ Completely pass  12. Do you have enough money? □ Not enough □ Rarely enough □ Enough (average) □ Most enough □ Completely enough  13. Do you have all the information you need in your daily life? □ Not at all ready □ Rarely ready □ Ready (normal) □ Most ready □ Completely ready  14. What is your ability to act? □ Very bad □ Bad □ Not good and not bad □ Good □ Very good  15. Are you satisfied with your sleep? □ Not satisfied □ Not satisfied □ Neither satisfied nor dissatisfied □ Satisfied □ Very satisfied  16. Are you satisfied with your ability to do the things of daily life? □ Not satisfied □ Not satisfied □ Neither satisfied nor dissatisfied □ Satisfied □ Very satisfied  17. Are you satisfied with yourself? □ Not satisfied □ Not satisfied □ Neither satisfied nor dissatisfied □ Satisfied □ Very satisfied  18. Are you satisfied with the support you have received from your friends? □ Not satisfied □ Not satisfied □ Neither satisfied nor dissatisfied □ Satisfied □ Very satisfied  19. Are you satisfied with the ease of access to health care? □ Not satisfied □ Not satisfied □ Neither satisfied nor dissatisfied □ Satisfied □ Very satisfied  20. Do you have negative feelings? (eg low mood, despair, anxiety, depression)  □ No negative feelings □ Sometimes negative feelings □ Sometimes negative feelings □ Often negative feelings □ Always negative feelings |

# 8 Safety evaluation

During the trial period, the safety of the study drug was evaluated through adverse event records (including serious adverse events), laboratory tests, vital signs, physical examination, echocardiography and electrocardiogram records. During the trial, the symptoms and signs of the subjects after the drug should be closely observed. Adverse events/reactions that occur should be dealt with in a timely and effective manner to ensure the safety and interests of the subjects. After the occurrence of adverse drug events/reactions has been dealt with in a timely and effective manner, the type, symptoms, time of occurrence, degree (or grade), symptomatic treatment methods and outcomes should be recorded, and then the adverse events should be analyzed, assessed, and counted as the basis for the continuation of the test.

# 9 Case dropout

All subjects who have filled out the informed consent form and are screened to be eligible to enter the trial have the right to withdraw from the clinical trial at any time. No matter when and for any reason, as long as the subjects who have not completed at least one trial drug administration and cannot be evaluated for safety and efficacy, they are dropout cases (disregarded due to disease progression and clear medical evidence after enrollment). to fall off). When a subject drops out, the researcher must record the reason for the drop out, complete the assessment items that can be completed, and fill in the last visit record carefully. For those who fall off due to adverse reactions and are finally judged to be related to the trial drug after follow-up, the investigator should be notified. Subjects who dropped out of the study after screening without obtaining a drug number were not considered dropout cases. If subjects completed a complete cycle and have detailed records, statistical analysis should be performed in the safety evaluation.Subjects who withdraw from the study cannot be re-entered and their number cannot be reused.
